# Evisceration of gallbladder at the site of a Pezzer drain: a case report

**DOI:** 10.4076/1757-1626-2-8601

**Published:** 2009-07-31

**Authors:** Bayrak Vedat, Sumer Aziz, Kotan Çetin

**Affiliations:** 1 Department of Surgery, Faculty of Medicine, Yüzüncü Yil UniversityVanTurkey; 2 Department of Surgery, Kaş State HospitalAntalyaTurkey

## Abstract

**Introduction:**

The drainage of abdominal cavity by means of tube drains is the oldest method. The herniation of gallbladder through the abdominal wall is very rare. Although there are studies informing evisceration of organs such as small intestines and ovary from drain site; at the literature scanning, no publication has been met with advising gallbladder evisceration from the Pezzer drain site so far.

**Case presentation:**

We describe here the first case in the literature of gallbladder evisceration from the Pezzer drain site. A male case with a history of operated incarcerated right inguinal hernia presented with a surgical abdomen. With a diagnosis of intestinal ischemia, the patient underwent laparotomy. About 200 cc fluid with serous quality has been determined in the abdomen and aspirated. Patchy ischemia zones were observed in ileum, over serous face. No. 30 Pezzer’s drain was placed for the intention of drainage, extending from the right side of navel towards rectovesical area. After the drainage stopped patient was discharged from hospital with recovery on the 8^th^ postoperative day. One day after the discharge, applied to another general surgery center, by complaining that the intestine protruded through an opening in its surrounding walls of the drain place and that has been gradually enlarged. During the operation it was determined that proximal 2/3 of gallbladder has been protruded to outside of abdomen. Cholecystectomy was performed, and patient recovery was uneventful.

**Conclusions:**

Gallbladder herniation is a pathology requires emergency operation.

## Introduction

Drainage is a method which is used to prevent noxious materials from building up in the abdominal cavity. Application of drains has been known for more than 2000 years. Recently, parallel to the developments in surgical techniques and technological advances, surgical abdominal procedures have become safer with less postoperative complications. Draining abdominal cavity by drainage tubes is the oldest method [1]. However, it should be known that their effect proves to be minimal because as a result of the accumulation of fibrin within lumen, they rapidly get clogged and become dysfunctional. Moreover, drains placed into the peritoneum cause erosions on walls of organs or vessels which lead to fistula formation, and eventually elevate the risk of hemorrhage. Drains result in foreign body reaction in the organism and induce disruption of neutrophil functions and elevation of infection risk [2]. Drains are usually employed for prophylactic purposes aiming to provide information on wound status and drain potential collections in presence of diffuse peritonitis, and used for drainage and cleaning purposes in the presence of localized fluid collections and abscesses. Currently, while drains are commonly employed in surgical interventions, benefits of drainage have not been shown in experimental models. Indications of drain usage have not been clearly defined and remains to be a contentious field. Lately, indications of drain usage following various surgeries, have been started to be questioned. However, therapeutic drain usage resumes to bear importance particularly for surgical procedures performed on lesser pelvis and surgical operations involving major bacterial contamination [1-3].

While there are studies that report evisceration of organs such as small intestine and ovary at the site of a drain, no study which involved evisceration of a gallbladder at the site of a pezzer drain, has been found in the literature [4,5].

The aim of the present study was to evaluate our patient who experienced evisceration of gallbladder at the site of a Pezzer drain, which is believed to be the first case in the literature, in light of the related literature data.

## Case presentation

A 56-year-old Turkish male patient had been diagnosed with incarcerated inguinal hernia after presenting to a different general surgery center due to swelling in the right groin. Because of the anesthesiologist’s decision to evaluate him as ASA E due to pulmonary problems, he had been operated under local anesthesia. Perioperatively, ischemic alterations had been observed in the incarcerated small intestine loop. Intestinal viability had not been evaluated completely. Monitoring of the patient had been decided prior to application of resection, and following the reduction of the intestinal loop back into the abdomen, herniography had been applied with propylene mesh. The case had been referred to the Emergency Surgery Department of our teaching and research hospital due to development of abdominal distension, presence of air-fluid levels on erect abdominal direct radiographs (EADR), and absence of gas-feces expulsion during the post-operative period. We evaluated the patient in Emergency Service. Laboratory results showed normal CBC values. Biochemical parameters were as follows: CK : 794 U/L (0-270), AST : 72 U/L (0-38), LDH : 555 U/L (240-480), Ca : 7.2 mg/dl (8.4-10.3). EADR of the patient displayed air-fluid levels on the abdomen. Abdominal ultrasonography (US) showed considerably dilated intestinal loops, intestinal content moving to and fro, and grade 2 bilateral renal parenchymal disease.

**Figure 1.**
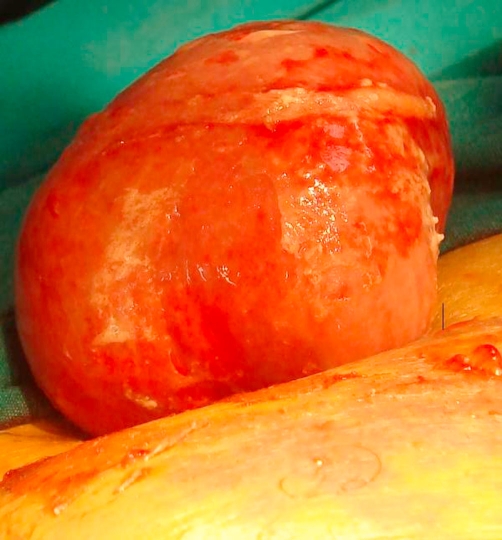
The view of mass, which can not be reduced from the drain place and resembles intestinal ans.

**Figure 2.**
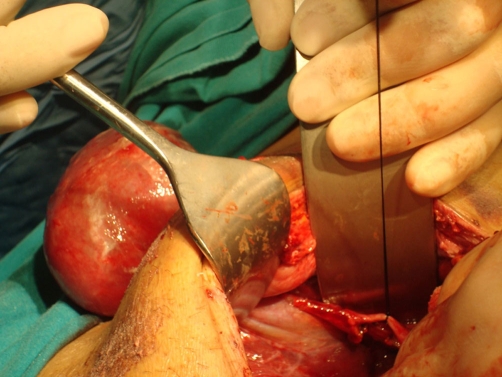
The view of protruded gallbladder.

**Figure 3.**
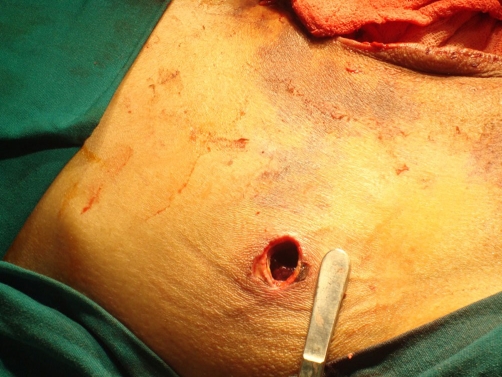
The view of drain place diameter.

Laparotomy was performed due to consideration of intestinal ischemia. Serous fluid of an amount of 200 cc was found in the abdomen and aspirated. Serosal surface of the ileum exhibited ischemic areas in patches. Lembert 3.0 silk sutures were applied to those areas. Pezzer drain (No. 30) was placed from the right portion of the abdomen extending through rectovesical site. Feces evacuation was observed on the postoperative 3rd day. After reaching the amount of 20 cc drainage, the drain was removed on the postoperative 5th day. Following removal of the drain, around 20-30 cc serous discharge was observed daily from the drain site. Abdominal distension was present until the postoperative 6th day. The case which had shown expulsion of gas and feces, exhibited a reduction in distension followed by total resolution. The case showed no discharge from the drain site on postoperative 8th day and discharged in good health. One day after the discharge, our patient applied to another healthcare center with the complaint of a herniated intestine from his drain site which gets larger by time. He was referred to our clinic from this center with the prediagnosis of intestinal herniation. Initial evaluation in Emergency Service revealed absence of evacuation of feces and gas. The EADR taken showed gas accumulation but no fluid-air levels. A tissue of 5 × 4 × 3 cm, which was irreducible, edematous, and appearing like ischemic and similar to an intestinal loop, was determined to be protruding from the drain site (Figure 1). He was operated with the diagnosis of intestinal herniation at the drain site. During the operation, proximal 2/3 of the gallbladder was determined to be protruding out of the abdomen from the drain site (Figure 2). Mesentery of the gallbladder was intact. Hilar cholecystectomy was conducted and the gallbladder was removed. Diameter of the drain site was observed to be around 1 cm (Figure 3). Eventually the drain site was closed one by one from inside the abdomen with 1.0 silk sutures.

## Discussion

According to the search we performed in the medical literature, evisceration of gallbladder at the site of a drain, is the first case in whole medical literature. Moreover, there was only one case of incisional hernia including gallbladder herniation which was reported by Benzoni et al. [6]. Garcia et al. [7] described a case of parastomal hernia involving gallbladder herniation. Sirikci et al. [8] published a case of a pregnant woman with incisional hernia including gallbladder hernia. Furthermore, Shirahama et al. [9] reported a gallbladder herniation at the site of right subcostal incision due to splenomegaly caused by gallbladder carcinoma and liver cirrhosis. Carragher et al. [10] reported a dilated gallbladder herniation case associated with cholecystocutaneous fistula. In the present case, we determined a gallbladder protruding out of the abdominal wall from the Pezzer drain site.

It is contentious if this case should be identified as an evisceration or an incisional herniation. While several authors describe protruding of intraabdominal organs out of the abdomen as evisceration, some call it as hernia [11,12]. Evisceration is described as removal of organs as a result of the partial or complete separation of the operative incision which has been closed by several layers. Incisional hernia is identified as herniation of intraabdominal or preperitoneal structures due to poor or late healing of incisions on the anterior abdominal wall. In the present case, because there was no poor or late healing of incisions on the abdominal wall, the term incisional hernia remains inadequate. On the other hand, since there was no wound healing or separation of layers, the term evisceration fails to describe the case, as well. However, we preferred to call the incident, as an evisceration.

In such cases, the first thing one would suggest as the underlying etiology, may be hypermobile gallbladder attached to the liver by mesentery or a gallbladder longer than normal. Hypermobile gallbladders attached to the liver by a long mesentery, create a predisposition for torsion and incarceration. In the literature, while there has been one case exhibiting incarcerated gallbladder in ventral hernia [13], another case in the literature has displayed the same in spigelian hernia [14]. A case, in which gallbladder was determined even in inguinal hernia sac [15]. In the present case, gallbladder mesentery was fixated to the liver until the diaphragmatic surface of the liver. However, fundus of the gallbladder was found to be longer than normal and continuing for a distance of 7 cm after the gallbladder bed. That may be a factor causing protrusion of the gallbladder.

Another possible factor may be the large drain site. A case showing small intestine evisceration has been reported in the literature [11]. However, in the present case, drain site had a diameter of approximately 1 cm and had been opened minimally just as to allow placement of a Pezzer drain (No. 30).

Increased intraabdominal pressure may be another underlying cause of gallbladder evisceration [13]. Our case developed a temporary adynamic ileus during the postoperative period. While this incident seems to be a result of the elevated intraabdominal pressure, patient had exhibited gas and feces expulsion and had been discharged uneventfully. Moreover, there was no action that might supposedly and abruptly increase the intraabdominal pressure such as coughing or straining. Undoubtedly, elevated intraabdominal pressure can be considered as a predisposing factor for protrusion. However, the fact that instead of small intestine which is a more mobile structure in nature, gallbladder was the protruding organ, and that protrusion had occured in the drain site, all make this case more uncommon and interesting.

The reason behind confusion in preoperative diagnosis, was the absence of a case with a gall bladder evisceration at the site of the drain in the literature, and similarity of the morphologic appearance of edematous and ischemic gallbladder wall and edematous and ischemic small intestine wall. The intense gas accumulation was evaluated as an early period sign of intestinal herniation which caused confusion in preoperative hollow organ injury diagnosis. Immediate surgical intervention bears importance in such cases due to possible strangulation incidents.

## Conclusions

While gallbladder herniation is a serious pathology similar to intestinal herniation which requires immediate intervention, correct preoperative diagnosis can make a difference in the preferred treatment option such as deciding to employ laparoscopic cholecystectomy.

## Abbreviations

ASA: American Society of Anesthesiologists
AST: Aspartate aminotransferase
CBC: Complete blood count
CK: Creatinekinase
EADR: Erect abdominal direct radiography
LDH: Lactate dehydrogenase
US: Ultrasonography
